# Direct SERS tracking of a chemical reaction at a single 13 nm gold nanoparticle†
†Electronic supplementary information (ESI) available. See DOI: 10.1039/c8sc04496a


**DOI:** 10.1039/c8sc04496a

**Published:** 2018-12-04

**Authors:** Kun Zhang, Yujie Liu, Yuning Wang, Jingjing Zhao, Baohong Liu

**Affiliations:** a Department of Chemistry , Shanghai Stomatological Hospital , State Key Lab of Molecular Engineering of Polymers, and Collaborative Innovation Center of Chemistry for Energy Materials , Fudan University , Shanghai 200433 , China . Email: bhliu@fudan.edu.cn

## Abstract

Metal nanoparticles (NPs) with decreased sizes are promising catalysts in energy and medicine. Measuring the local reactions and simultaneously acquiring molecular insights at single small NPs, however, remain an experimental challenge. Here we report on surface-enhanced Raman spectroscopic (SERS) tracking of catalytic reactions of single 13 nm gold NPs (GNPs) *in situ*. We designed spatially isolated (>1.5 μm of inter-dimer space) GNP dimers, each of which consisted of two GNPs with sizes of ∼200 and ∼13 nm, respectively. This design integrates the SERS and catalytic activities into a single entity, while eliminating the crosstalk between adjacent particles, which allows us to trace the redox-derived spectral evolution at single 13 nm GNPs for the first time. We also quantified the reaction kinetics of each individual GNP and analyzed the average behavior of multiple GNPs. There is a large variability among different particles, which underscores the significance of single particle analysis.

## Abstract 2

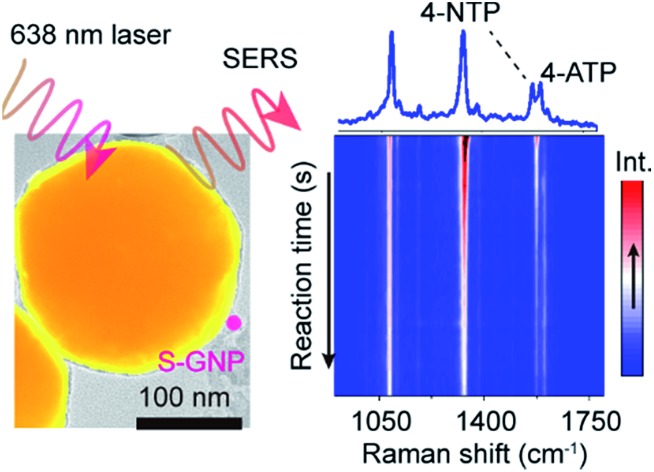
 *In situ* surface-enhanced Raman spectroscopic (SERS) probing of the local catalytic reaction at single 13 nm gold nanoparticles is reported.

## Introduction

Metal nanoparticles (NPs) such as gold NPs (GNPs) have been studied and explored in a wide range of fields from chemical synthesis and energy conversion to biochemical sensing because of their unique properties, especially their promising catalytic activities.^1^–^5^ Previous studies revealed that the catalytic activity of NPs is highly dependent on their structural characteristics such as the shape and size.^6^–^8^ With decreasing particle size, the NPs generally exhibit superior or new catalytic properties due to the increased surface-to-volume ratios and chemical potentials.^4^ The traditional ensemble techniques provide only averaged properties of NPs with varied shapes and size in a sample. Investigating the surface catalytic reactions at single NPs is thus essential to analyze the relationship between the structure and catalytic activity of the NPs. Studying chemical transformation at the single entity level has been achieved by single-molecule fluorescence microscopy,^9^–^11^ scattering spectroscopy based on dark imaging microscopy (DFM),^12^ electrochemiluminescence,^13^,^14^ plasmonics-based electrochemical current microscopy,^15^–^17^ or scanning probe microscopy.^18^,^19^ These techniques provide high throughput, sensitivity and spatial resolution but at the expense of structure information of molecules. Moreover, due to the limit of CCD sensitivity, conventional DFM is only adapted to large GNPs (L-GNPs) with dimensions over 50 nm, which is often catalytically inactive.

Surface-enhanced Raman spectroscopy (SERS) is a surface-specific technique that can provide structural “fingerprints” of molecules adsorbed on noble metal nanostructures.^20^ As such, extensive effort has been made to study NP-catalyzed chemical reactions by SERS *via* the construction of a bifunctional platform combining both the plasmonic and catalytic activities.^21^–^27^ Recently, SERS-based investigations of plasmon-driven photocatalysis at the single micro(nano)particle and even single molecule level have been achieved.^28^–^31^ In spite of these research progresses, the direct observation of catalytic processes occurring on a single small GNP (S-GNP) catalyst (*d* < 15 nm) in solution phase by SERS has not been demonstrated. This is mainly caused by the following aspects: (1) the insufficient plasmonic activity of the small catalyst NPs; (2) the more complexed measurement environment at a liquid–solid reaction interface; and (3) the low spatial resolution to the micrometer scale of SERS due to the diffraction limit of light.

**Scheme 1:**
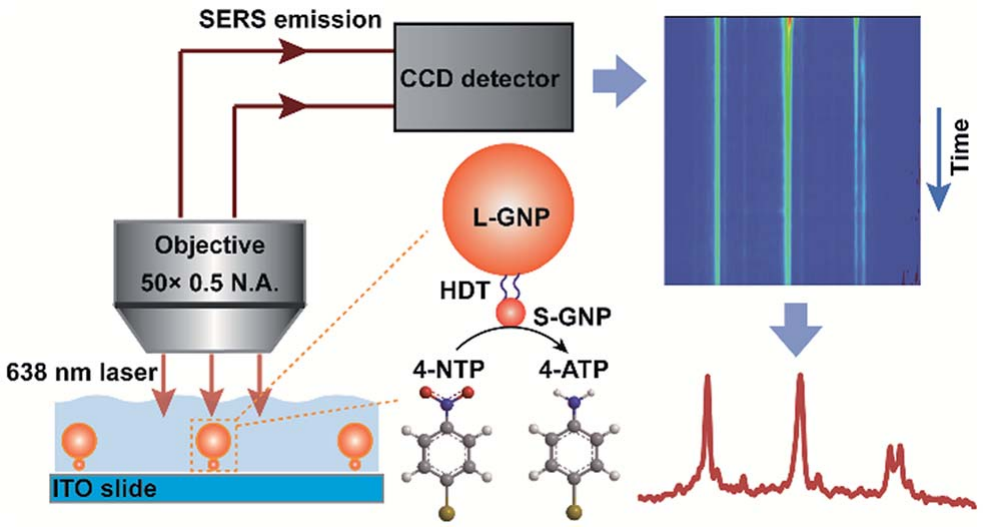
Illustration of *in situ* SERS tracking of catalytic reactions at single 13 nm GNPs. Spatially isolated dimers, each of which consists of a single L- and S-GNP bridged by 1,6-hexanedithiol (HDT), are electrostatically immobilized on the indium tin oxide (ITO) glass surface. 4-Nitrothiophenol (4-NTP) is chemically reduced to 4-aminothiophenol (4-ATP) under the catalysis of a single 13 nm S-GNP. The accompanying Raman vibrational changes are enhanced by the electromagnetic field of a 200 nm L-GNP and detected by the Raman microscope.

Here we report, for the first time, the direct SERS observation of the chemical transformation catalyzed by a single 13 nm S-GNP in aqueous solution (Scheme 1). To overcome the above limitations, we designed and prepared well-isolated S-GNP-molecule-L-GNP dimer nanostructures with three components in the molecular layer, a reactive molecule as the reaction substrate, an inert molecule as a Raman inner standard, and a linker molecule to form the GNP dimers. The hydrogenation of 4-nitrothiophenol (4-NTP) in the presence of sodium borohydride, a promising model reaction for SERS characterization of the catalyst performance,^21^–^23^,^25^,^27^ was studied at the molecular level. We use 1,6-hexanedithiol (HDT) to serve as the molecular bridge because of its bifunctional property, appropriate molecular length (1 nm),^28^ and small Raman cross-section. Moreover, since only HDT is involved in the assembly of the GNP dimers, studying other types of reactions such as the decarboxylation of 4-mercaptobenzoic acid or the oxidation of phenylboronic acids is potentially feasible with the proposed SERS strategy. We extracted the dynamic spectral information during the reduction of 4-NTP at single S-GNPs, quantified the corresponding reaction kinetics, compared the catalytic reactivity between different GNPs, and demonstrated the heterogeneity in reactivity of individual S-GNPs.

## Results and discussion

The spatially isolated GNP dimers were prepared through the stepwise assembly of Au particles with different sizes on indium tin oxide (ITO) glasses (Scheme S1†).^32^ The ITO slides were first silanized with 3-aminopropyltrimethoxysilane (APTMS), followed by immersing into the 13 nm S-GNP suspension (Fig. S1a†) to immobilize the nanocatalysts *via* electrostatic interactions. After removing all the amine functional groups from the ITO surface except those binding with the S-GNPs, the slides were put in an ethanol solution consisting of 4-NTP, 2-naphthalenethiol (2-NT) and HDT to functionalize the S-GNPs. Finally, the GNP dimers were formed by chemical adsorption of the 200 nm L-GNPs (Fig. S1b†) with HDT (the experiment details were provided in the ESI S1†).

**Fig. 1:**
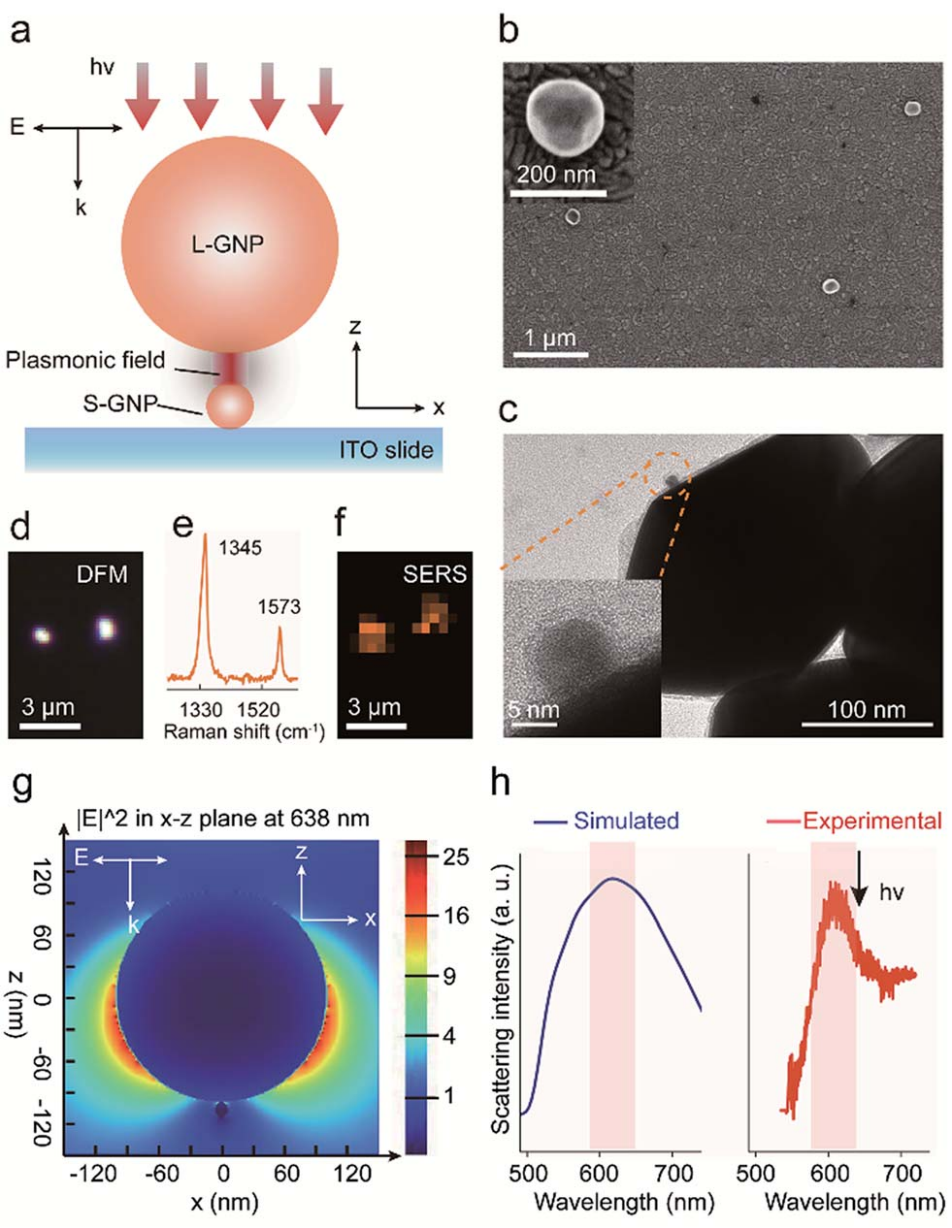
(a) Schematic diagram showing the configuration of a L-GNP/S-GNP dimer linked with 1,6-hexanedithiol. (b) SEM and (c) TEM images of the dispersed GNP dimers. The insets show a magnified view of a typical GNP dimer. (d) DFM image of two isolated GNP assemblies. (e) SERS spectrum of 4-NTP measured from an isolated GNP dimer. (f) SERS image of the same assemblies shown in (d), constructed by using the intensity of the 1345 cm^–1^ peak of 4-NTP in (e). (g) FDTD calculation showing the local electric field distribution of the GNP dimer at the *X*–*Z* plane. (h) Scattering spectrum of a single GNP dimer.

We systemically characterized the preparation of the GNP dimer nanostructures. The scanning electron microscopy (SEM) measurement indicated that the S-GNPs are well dispersed on the ITO surface (interspaces ≥1.5 μm) and most of them exist as monomer with a yield as high as 96% (Fig. S2†). As the L-GNPs are ∼15 times larger than the S-GNPs, the SEM images taken after performing the assembly operation provide only the feature of the L-GNPs (Fig. 1b and S3†). To verify successful preparation of the GNP dimer structures, we detached the nano-assemblies from the ITO surface by sonication and detected them by transmission electron microscopy (TEM). As shown in Fig. 1c and S4,† each asymmetric nano-assembly consists of two GNPs with different sizes at ∼13 and ∼200 nm, respectively. The GNP dimers are well dispersed on the ITO surface with an average inter-dimer space of 1.95 ± 0.76 μm (Fig. 1b and S3†). These results imply that optical emissions from individual GNP dimers can be distinctively detected by the objective of our confocal Raman microscope with a spot size of ∼1.56 μm. This is validated by the high quality SERS spectrum of 4-NTP obtained from a single GNP dimer upon excitation with 638 nm laser (Fig. 1e and S5.† See peak assignment in Table S1†). With the aid of DFM (Fig. 1d), we imaged two adjacent GNP dimers and observed a fairly strong SERS emission from each single S-GNPs which could be distinguished with each other (Fig. 1f, intensity map of the 1345 cm^–1^*ν*_(NO)_ peak. See also Fig. 1e). No SERS signature was observed in the absence of 4-NTP because of the small Raman cross-sections of HTD as described above (Fig. S6†). To understand the underlying physical mechanism of Raman enhancement, we used the finite-difference time-domain (FDTD) method to model the local electromagnetic field distribution of the GNP dimer.^33^,^34^ As shown in Fig. 1g, the plasmonic electric field is strongly enhanced both at the L-GNP surface as well as the gap junction between the L- and 13 nm S-GNPs when the sample is excited by a forward and vertical 638 nm laser beam, the same parameter used in the SERS measurements. Of note, the choice of 13 nm GNPs as S-NPs is a compromise between catalytic activity and plasmon-induced electric field enhancement (see part 3 and Fig. S7†). The GNP dimer shows a broad scattering wavelength centered at ∼620 nm, which resonates with the focused laser beam at 638 nm (Fig. 1h).

**Fig. 2:**
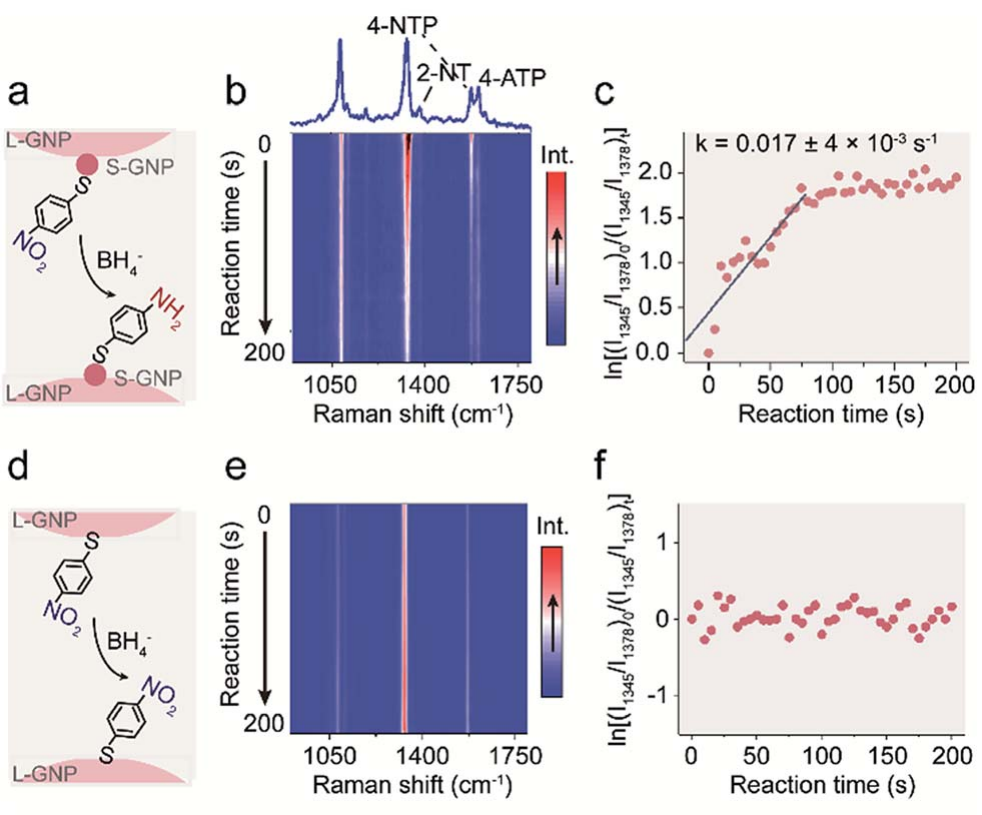
(a) Schematic illustration showing the S-GNP catalyzed reduction of 4-NTP in the presence of borohydride. (b) Colour-coded intensity map of time-dependent single-NP SERS spectra after borohydride addition, with a range of Raman shifts between 900 cm^–1^ to 1800 cm^–1^ for a 1 s integration time, taken every 5 s at 638 nm. (c) Plot of ln[(*I*_1345_/*I*_1378_)_0_/(*I*_1345_/*I*_1378_)_*t*_] *versus* time for the determination of the rate constants for the reduction process with spectra shown in (b). (d) Schematic illustration and (e) colour-coded intensity map of time-dependent single-NP SERS spectra without borohydride addition. (f) Plot of ln[(*I*_1345_/*I*_1378_)_0_/(*I*_1345_/*I*_1378_)_*t*_] *versus* time.

Having the bifunctional GNP dimer in hand, we first investigated the capability of this nano-platform in real-time probing of the catalytic processes at a single S-GNP. To this end, the S-GNP surface was co-functionalized with a mixture of 4-NTP and 2-NT. 2-NT does not react with sodium borohydride, thus is used as an internal standard for relative quantification. Fig. 2b shows the time-resolved SERS spectra obtained from a single GNP dimer structure after the addition of borohydride aqueous solution. Initially, the spectrum exhibited typical signatures of 4-NTP and 2-NT (Fig. S8 and S9†) including the *ν*(CS) at 1080 cm^–1^, *ν*(NO_2_) at 1345 cm^–1^, *ν*(CC) at 1573 cm^–1^ for 4-NTP (Table S1†) and the *ν*(CC) at 1378 cm^–1^ for 2-NT (Table S2†). Then, the intensity of the 4-NTP associated bands decreased gradually with the simultaneous appearance and increase of a new band at 1590 cm^–1^ (Fig. 2b, S8 and S9†), assigned to the *ν*(CC) of 4-ATP (Table S3†), indicating the transformation of 4-NTP to 4-ATP (Fig. 2a). Since the borohydride aqueous solution we used is buffer-free, a near-neutral pH environment is expected in our reaction system. According to a recent research by Xie *et al.*, the reduction is mainly driven by the borohydride-derived molecular hydrogen (H_2_) at this condition.^27^ To quantitatively study the reaction dynamics, the intensity of the 1345 cm^–1^ 4-NTP band, relative to the 1378 cm^–1^ band intensity of 2-NT was extracted as a function of time. The relative SERS intensity (ln(*I*_1345_/*I*_1378_)) trajectory is plotted in Fig. 2c. As an obviously excessive dose of borohydride is used, its concentration is assumed to stay constant during the catalytic process (estimation of the molar ratio between 4-NTP and borohydride is shown in the ESI S2†). The reaction is thus assumed to follow the pseudo-first-order kinetics, and the rate constant (*k*) is given by eqn (1):1
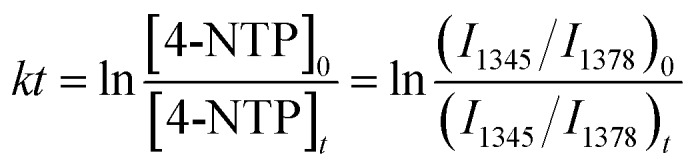
where *I*_1345_ and *I*_1378_ are intensities of the SERS peaks at 1345 and 1378 cm^–1^, respectively. From the above linear model, the catalytic rate constant was measured to be 0.017 ± 4 × 10^–3^ s^–1^. As a control, we carried out the experiment using GNP dimers prepared as above but without the addition of borohydride in aqueous media, which did not show any detectable change in spectrum during the trajectory (Fig. S10†). We also performed an additional control experiment with 4-NTP functionalized L-GNP monomers in the presence of borohydride, which did not show a change in spectrum either (Fig. 2d–f). These control experiments indicate that both the S-GNP and borohydride play key roles in the reduction process.

**Fig. 3:**
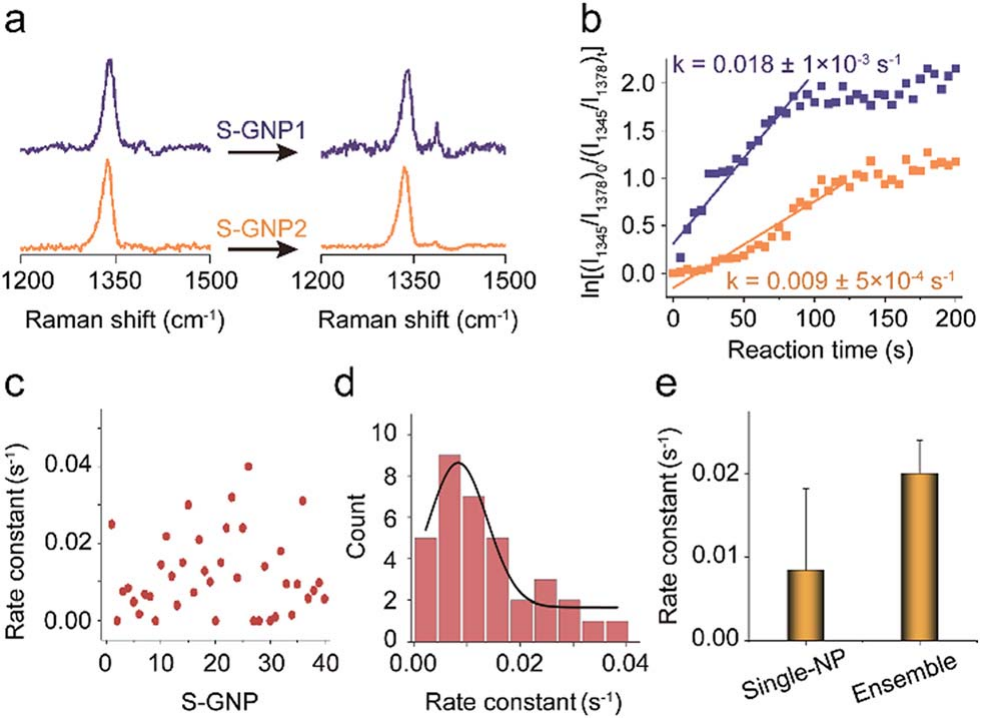
(a) SERS spectra corresponding to two different catalysts discussed in the main text, S-GNP1 and S-GNP2, before (0 s) and 60 s after the addition of borohydride for a 1 s integration time, taken every 5 s at 638 nm. (b) Plot of ln[(*I*_1345_/*I*_1378_)_0_/(*I*_1345_/*I*_1378_)_*t*_] *versus* time for the determination of the rate constants for the reduction process with the S-GNPs shown in (a). (c) Rate constants obtained from 40 S-GNPs and (d) their statistical distribution. (e) Comparison between the average rate constant obtained from the single nanoparticle measurement with that from the ensemble measurement by assembling the densely packed S-GNPs on the amino functionalized Si wafer.

As discussed in the introduction section, the physical and chemical properties of nanoscale materials are structurally dependent.^6^–^8^,^35^–^38^ From the TEM image, although the S-GNPs exhibit a relatively narrow size distribution, they are actually faceted rather than perfect spherical in morphology (Fig. S1†). In order to examine if such fluctuations would lead to differences in their catalytic activities, the catalytic events occurred at two different S-GNPs were measured. Fig. 3a shows the corresponding SERS spectra before and after the addition of borohydride (60 s). The intensity ratio *I*_1345_/*I*_1378_ for the S-GNP2 is obviously lower than that measured from the S-GNP1, implying different reaction kinetics at the two S-GNPs. This is also reflected by calculation of the reaction rate constants. As shown in Fig. 3b, the rate constant for the S-GNP1 (0.018 ± 5 × 10^–4^ s^–1^) was about 2 times of that for the S-GNP2 (0.009 ± 5 × 10^–4^ s^–1^). The difference in reaction dynamics may come from the intrinsic different catalytic abilities of the two particles or from the different molecular coverage at the two particles. We exclude the latter possibility by considering the following two aspects: first, the reactant 4-NTP contains one thiol group and has a circular cross-sectional area of about 0.8 nm-in-diameter,^39^ very close to the features of the internal standard 2-NT, so that a similar affinity to the gold surface is expected for the two molecules. Second, the S-GNPs are nearly monodispersed in diameter, as evidenced by the small deviation of ±2.7 nm. Third, we incubated the nanocatalysts in a millimolar level of 2-NTP/2-NT mixture solution overnight, leading to saturated adsorption at the catalyst surfaces. Therefore, the relative molecular density for 2-NTP and 2-NT should be constant over different catalysts, which has been confirmed by the research of Joseph *et al.*^21^Fig. 3c depicts the distribution of rate constants measured from 40 S-GNPs. A large variation in the reaction kinetics between each individual S-GNPs is observed. There is a statistical distribution in the rate constant for different S-GNPs. The distribution follows a Gaussian distribution, with an average rate constant of 0.0084 ± 5 × 10^–4^ s^–1^. Although the statistical rate value is general consistent with that obtained by the ensemble measurement, the later conceals the heterogeneity between single particles, which in tune underscores the significance of investigating the catalytic property in the single-entity resolution.

## Conclusions

We have demonstrated the direct SERS observation of surface catalytic reactions at single 13 nm S-GNPs in aqueous solution. By functioning each individual well-isolated S-GNPs with the molecular bridge of HDT and then coupled with a 200 nm L-GNP, we are able to integrate the SERS and catalytic activities into a single GNP dimer nano-assembly. That allows us to track the redox process at individual S-NPGs *in situ*, quantify the apparent rate constants for different nanocatalysts, analyze the average catalytic kinetics of multiple S-GNPs, and compare it with the ensemble measurement. The SERS trajectories and measured rate constants reveal a large particle-to-particle variability in the catalytic property, which is attributed to the structural heterogeneity in terms of the crystal morphology of different S-GNPs. To fully structural characteristics on the catalytic activities of single S-GNPs measured by SERS, future studies may benefit from combining SERS with high-resolution structural analytical techniques such as TEM, SEM and atomic force microscopy. By adjusting the components of the bifunctional dimer assemblies, we expect that the abilities of SERS demonstrated here are helpful in inspecting the intrinsic reactivity of single nanocatalysts and study the reaction pathway with molecular structural information.

## Conflicts of interest

There are no conflicts to declare.

## Supplementary Material

Supplementary information
