# Supplementary material for: Direct SERS tracking of a chemical reaction at a single 13 nm gold nanoparticle
Source: Chem Sci. 2018 Dec 4;10(6):1741–5. doi: 10.1039/c8sc04496a (PMC6374737; doi:10.1039/c8sc04496a)
Supplement: Supplementary file 1 [file SC-010-C8SC04496A-s001.pdf]

Electronic Supplementary Material (ESI) for Chemical Science.
This journal is © The Royal Society of Chemistry 2018

Electronic Supplementary Information for

Direct SERS Tracking of a Chemical Reaction at a Single 13-nm Gold Nanoparticle

Kun Zhang, Yujie Liu, Yuning Wang, Jingjing Zhao, and Baohong Liu*

*Corresponding author: bhliu@fudan.edu.cn

Table of Contents

S1 Experimental Procedures

S2 Estimation of the molar ratio between 4-NTP and borohydride

S3 Preparation and characterization of bifunctional GNP dimers

S4 Evaluation of plasmonic near field of gold dimer with a smaller GNP (8 nm) as catalyst

S5 SERS probing of surface catalytic reaction at a single GNP dimer

Section S1 Experimental Procedures

Reagents and Materials. All chemicals were of analytical grade, used without further purification, and obtained from commercial suppliers. Chloroauric acid ($HAuCl_4 \cdot 4H_2O$), sodium citrate ($Na_3C_6H_5O_7 \cdot 2H_2O$), and sodium borohydride ($NaBH_4$) were purchased from Sinopharm Chemical Reagent (Shanghai, China). 4-Nitrothiolphenol (4-NTP), 4-aminothiophenol (4-ATP), 2-naphthalenethiol (2-NT), and 3-aminopropyltrimethoxysilane (APTMS) were obtained from Sigma-Aldrich. Citrate stabilized gold nanoparticles (GNPs) with an average diameter of 200 nm were provided by BBI Solutions (Cardiff, UK). Indium tin oxide (ITO) conductive glasses (resistance ≤ 6 Ω/square, thickness 0.175 ± 0.05 mm) were purchased from Shenzhen Huanan Xiangcheng Technology Co., Ltd. (Guangdong, China). Purified water was provided by Hangzhou Wahaha Group (Zhejiang, China).

Synthesis of 13 nm GNPs. GNPs with an average diameter of 13 nm were prepared following the Turkevich/Frens citrate reduction technique.[1,2] In a typical synthesis, 50 mL of 1 mM $HAuCl_4$ was brought to a rolling boil under intense stirring. Rapid addition of 5 mL of 38.8 mM sodium citrate to the vortex of the solution resulted in a color change from pale yellow to burgundy. Boiling was continued for 10 min; the heating mantle was then removed, and stirring was continued until the solution was cooled to room temperature.

Preparation of GNP dimers. GNP dimers were prepared via a stepwise assembly technique as described by Yoon et al. with some modification.[3] ITO glasses were chosen as the substrates because of their good conductivity allowing us to facilely monitor the assembling process. Briefly, ITO slides (5 mm × 5 mm) were ultrasonically cleaned in acetone, ethanol, and ultrapure water for 15 min, respectively, followed by cleaning in H_2O/H_2O_2 (30%)/NH_4OH (5:1:1, v/v) for 30 min. The slides were further cleaned by washing with a large amount of water followed by drying at 60 °C for 30 min. The slides were immersed in an ethanol solution of APTMS (1%,

v/v) for 30 min for aminosilanization. Then, the slides were washed three times with ethanol followed by sonicating in ethanol for 5 min, and drying in an oven at 120 °C for 3 h. The amino-modified ITO slides were immersed in 5 mL of 10 pM S-GNPs solution for 5 min led to the adsorption of the catalyst NPs. After removing the residual GNPs by gently washing several times with water, the S-GNPs-adsorbed slides were placed in 5 mL of 1 mM NaOH aqueous solution for 5 h to remove all amino groups from the ITO surface except for those binding the S-GNPs. The slides were washed with water, dried at 60 °C, and then placed in 5 mL of 0.05 mM HDT ethanol solution containing 1 mM of 4-NTP and 0.2 mM of 2-NT for 12 h followed by washing with ethanol and drying on an electric heating plate. Finally, the slides were immersed in 5 mL of 1 pM L-GNPs solution for 2 h with gentle stirring led to the adsorption of the large particles on the surfaces of S-GNPs, forming GNP dimers. The slides were carefully washed with water and dried at 60 °C for further use.

Characterization. UV/vis absorption spectra were obtained on an Aglient HP8453 spectrophotometer. Scanning electron microscope (SEM) images were obtained on a Hitachi S-4800 filed emission scanning electron microscope. Transmission electron microscope (TEM) images were measured on an EOL JEM-2011 electron microscope. Raman experiments were performed on a Horiba XploRA confocal Raman microspectrometer. A 638 nm laser light was focused on the sample surface by means of a 50x Olympus objective lens (NA = 0.5), allowing the spot width to be ~1.55 μm on the illuminated location.

Section S2 Estimation of the molar ratio between 4-NTP and borohydride

The amount of borohydride in the liquid solution was calculated by Formula S1:

$$N_{borohydride} = c \cdot V \cdot N_A = 1\ \text{mM} \times 2.5\ \text{mL} \times 6.02 \times 10^{23} = 1.5 \times 10^{18} \qquad \text{(S1)}$$

where c is the concentration of the borohydride solution, V is the solution volume, N_A is Avogadro's constant.

According to the SEM measurements, the surface density of the S-GNPs on the ITO slide was estimated to be about 0.65 particles/μm^2. The ITO slide area (double surfaces) could be obtained with Formula S2:

$$S_{ITO} = 2a^2 = 2 \times (5\ \text{mm})^2 = 50\ \text{mm}^2 = 5\times 10^7\ \mu\text{m}^2 \qquad \text{(S2)}$$

where a is the side length of the iTO glass.

The total number of the S-GNPs in the reaction system was calculated to be 3.25×10^7 (N_{GNPs} = 0.65 particles/μm^2 × 5× 10^7 μm^2 = 3.25 × 10^7 particles). The surface area of a single 13 nm S-GNP was calculated by Formula S3:

$$S_{surf} = 4\pi r^2 = 4 \times 3.14 \times (6.5\ \text{nm})^2 = 530.66\ \text{nm}^2 \qquad \text{(S3)}$$

where r is the radius of the S-GNPs.

Thus, the total surface area of the S-GNPs in the reaction system could be obtained by $S_{total\text{-}GNPs}$ = 530.66 nm^2 × 3.25 × 10^7 = 1.725 × 10^{10} nm^2

The circular cross-sectional area for about 0.8 nm-in-diameter 4-NTP molecules was estimated to be 0.5 nm^2 according to Formula S4:[4]

$$S_{cs} = \pi r^2 = 3.14 \times 0.4^2 = 0.5\ \text{nm}^2 \qquad \text{(S4)}$$

Assuming only the 4-NTP molecules were assembled on the S-GNPs and formed a completely packed monolayer, the total number of the 4-NTP molecules assembled on the S-GNPs was about 3.45 × 10^{10} according to Formula S5:

$$N_{total\text{-}4\text{-}NTP} = S_{total\text{-}GNPs}/S_{cs} = 1.725 \times 10^{10}\ \text{nm}^2/0.5\ \text{nm}^2 = 3.45 \times 10^{10} \qquad \text{(S5)}$$

It is obvious that the amount of 4-NTP in the reaction system is far smaller than that of borohydride.

Section S3 Preparation and characterization of bifunctional GNP dimers

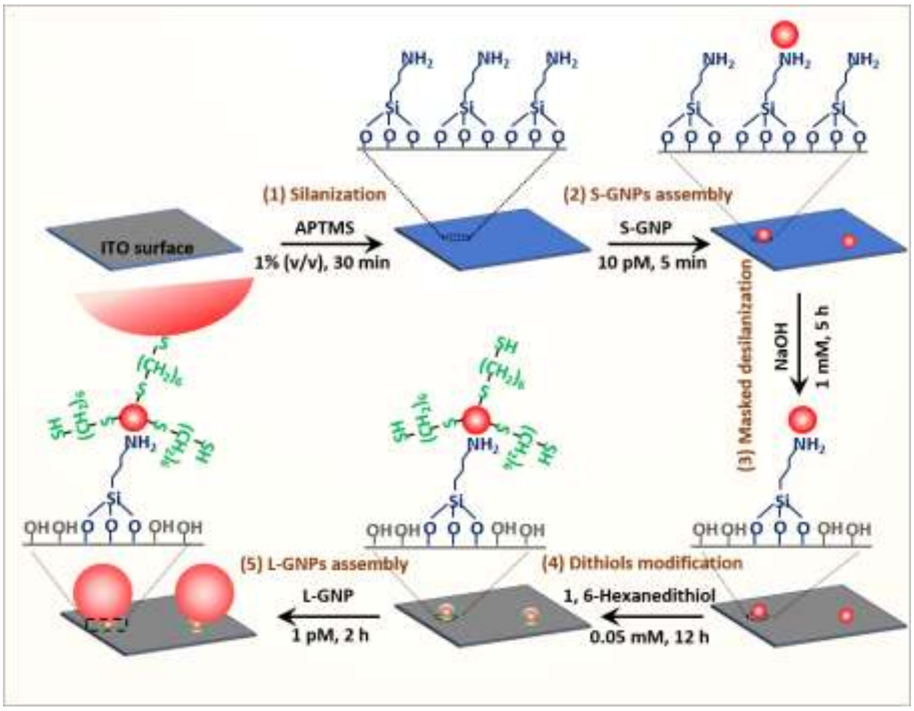

Scheme S1. Schematic illustration for stepwise dimer assembly using masked desilanization. (1) Amine coating of ITO slides using APTMS, (2) adsorption of S-GNPs, (3) masked desilanization with NaOH, (4) thiol-modification of S-GNPs with HDT, (5) preferential assembly of L-GNPs onto the surface of S-GNPs. **NOTE:** in practical experiments, a mixture of 4-NTP, 2-NT, and HDT with a given molar ratio was used in step (4). For simplicity, the two other components were not shown here.

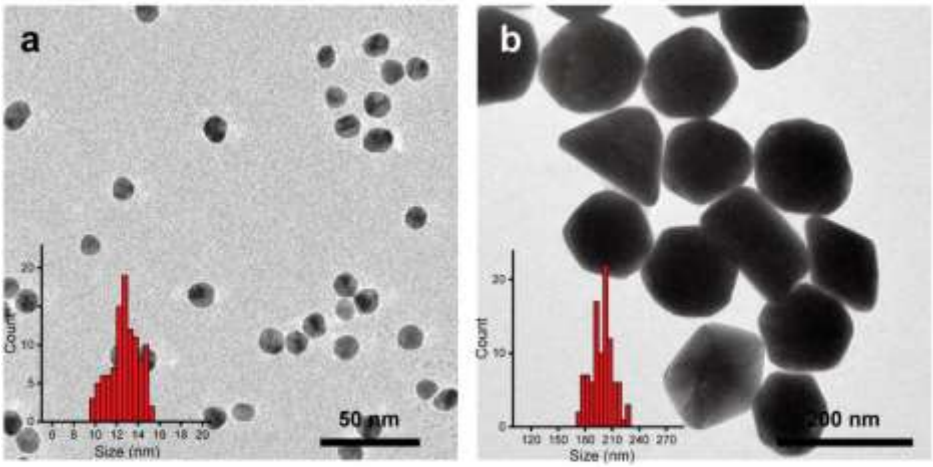

Figure S1. Representative TEM images of (a) the S-GNPs and (b) L-GNPs dried on a carbon film coated Cu-based grid. The average sizes for the S-GNPs and L-GNPs were found to be 12.8 ± 2.5 nm and 198.6 ± 12.4 nm. The variation in shapes were clearly observed for both types of nanoparticles. The size measurements were conducted on 200 particles for each types of GNPs.

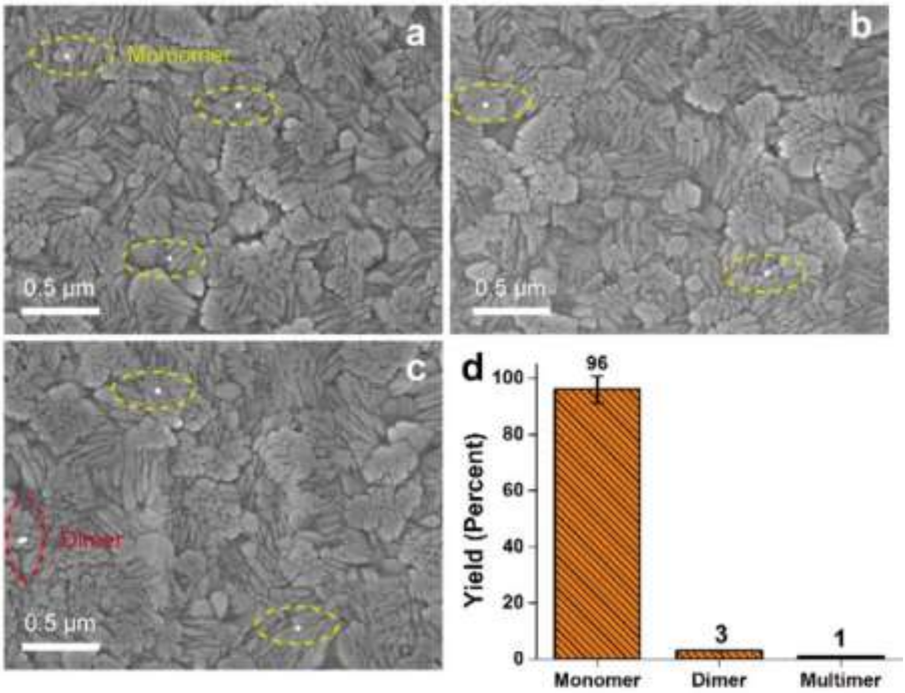

Figure S2. Representative SEM images showing the distribution of the S-GNPs on the surface of the ITO slide. (d) Statistics of the adsorbed S-GNPs on the ITO slides, measured from the SEM images of five different slides (total 120 particles).

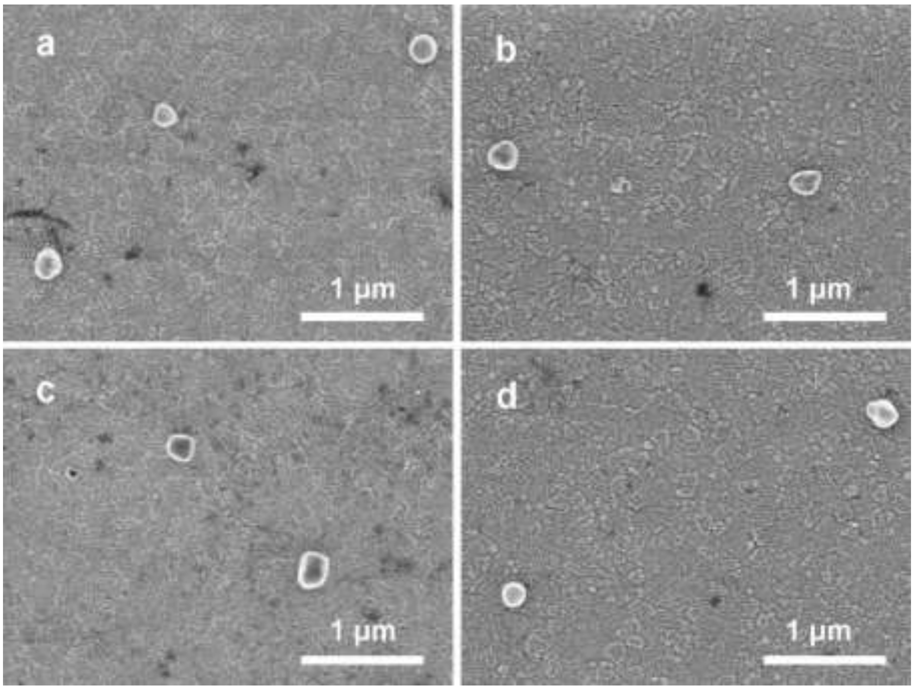

Figure S3. Representative SEM images showing the distribution of the GNP assemblies on the surface of the ITO slide. The average inter-particle distance was found to be ca. 1.95 ± 0.76 μm as estimated from total 30 particles.

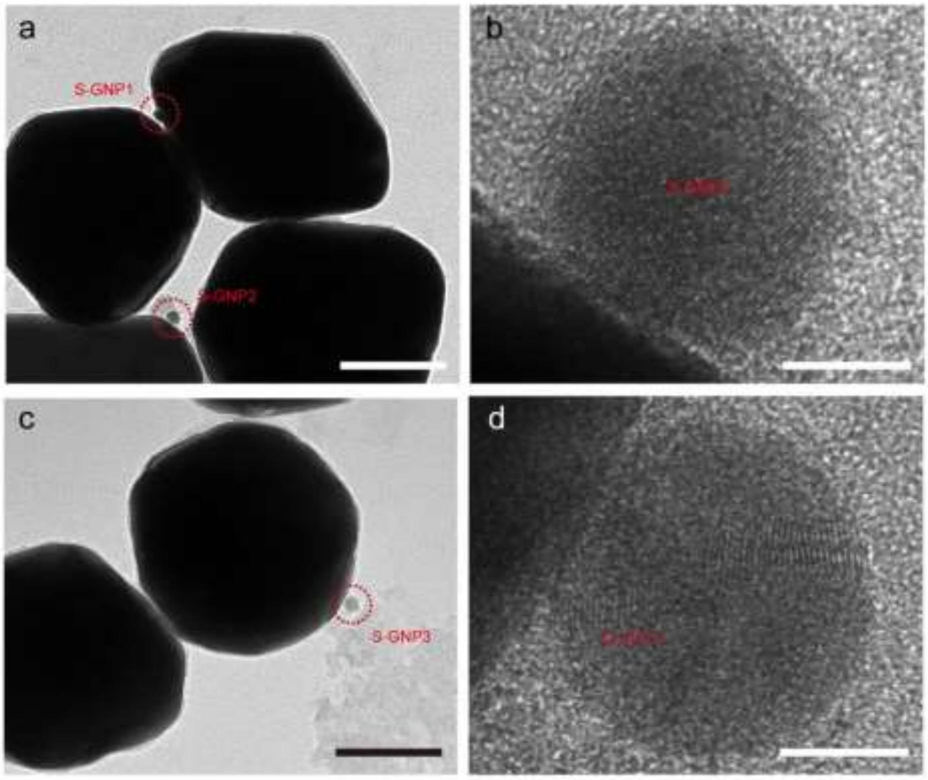

Figure S4. Representative TEM images of the L-GNP/S-GNP dimer assemblies. Images in (b) and (d) at higher magnification showing local areas labeled in (a) and (c), respectively. Scale bars represent 100 nm for (a) and (c), and 5 nm for (b) and (d).

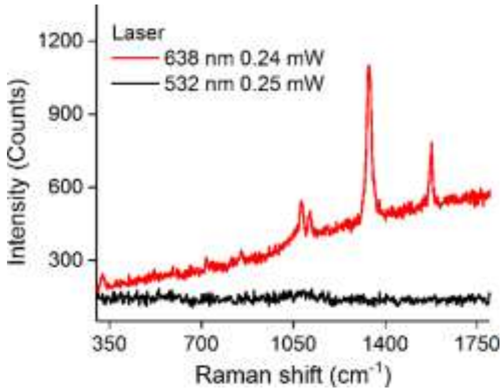

Figure S5. Laser wavelength-dependent SERS spectra of 4-nitrothiophenol (4-NTP) immobilized on the S-GNP surface and enhanced by the GNP dimer structure.

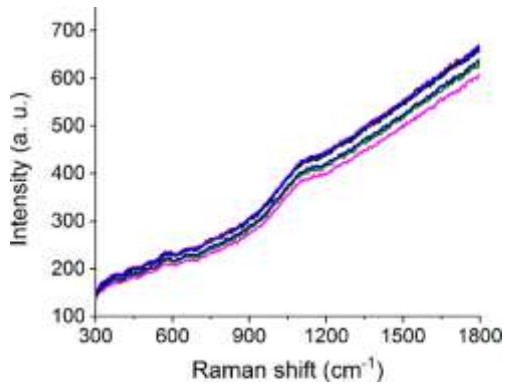

Figure S6. SERS spectra from the surfaces of the as-assembled GNP dimers showing no apparent background signals observed, probably because of the small Raman cross-sections of the linkers of HDT and APTMS.

Section S4 Evaluation of plasmonic near field of gold dimer with a smaller GNP (8 nm) as catalyst

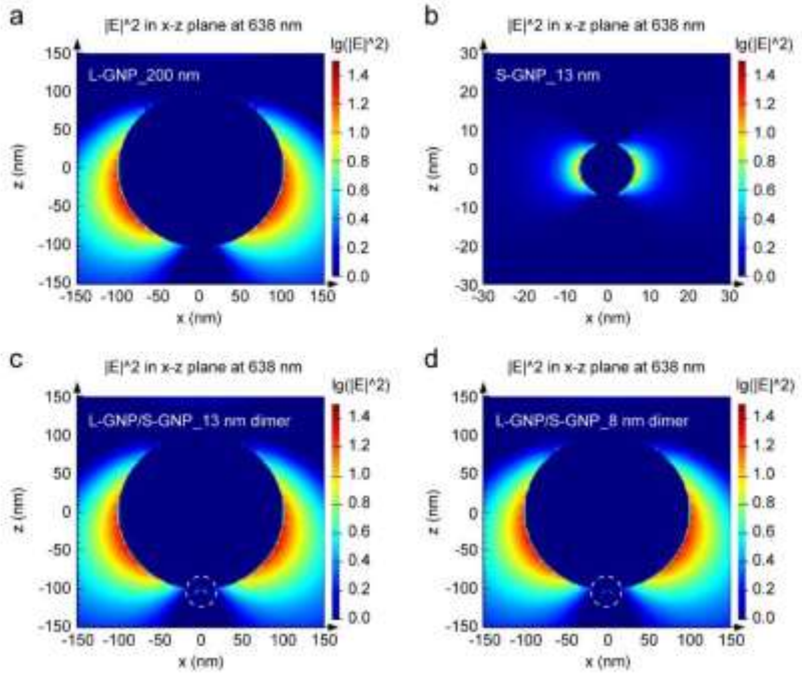

Figure S7. FDTD simulation of the local electric field distributions at 638 nm on single Au particle of (a) 200 nm or (b) 13 nm. The field distribution on Au dimers with single (c) 13-nm S-GNP or (d) 8-nm S-GNP as catalyst, respectively.

Figure S7a and be show that the local electromagnetic filed intensity near a single 200 nm-GNP is much stronger than that around a single 13 nm GNP under the excitation with 638 nm laser. If even smaller GNPs (8 nm) are used as S-NPs in the nanoassembly, the enhancement of local electromagnetic field is ca. 3 times lower as

shown in Figure S7c and d. In addition, as the particle diameter is decreased by F= d1/d2 (e.g., from 13 nm to 8 nm, F = 1.625), the surface area of the particle will decrease by $F' = (d1/d2)^2$ (here, $F' = 2.64$), leading to a reduction of the number of the molecules adsorbed also by a same extent of F′. As a result, the spectral intensity might be too weak to measure, especially for the product molecule of 4-ATP which has a smaller Raman cross section compared with 4-NTP. Considering this, we choose GNPs of 13 nm, which compromises the catalytic activity and the plasmon-induced electromagnetic fields enhancement.

Section S5 SERS probing of surface catalytic reaction at a single GNP dimer

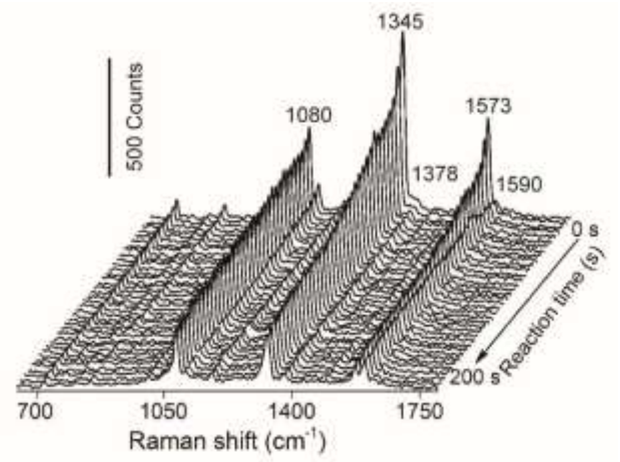

Figure S8. Time-dependent SERS spectra as shown in Figure 2a depicting the reduction of 4-NTP catalyzed by a single S-GNP.

Table S1. SERS vibrational modes of 4-NTP and their band assignments[5]

Raman shift (cm^{-1})	Band assignment
855	δ(CH)
1080	v(CS) and δ(CCC)
1110	δ(CH)
1345	$v(NO_2)$
1573	v(CC) and δ(CH)

v: stretching vibration, δ: deformation vibration

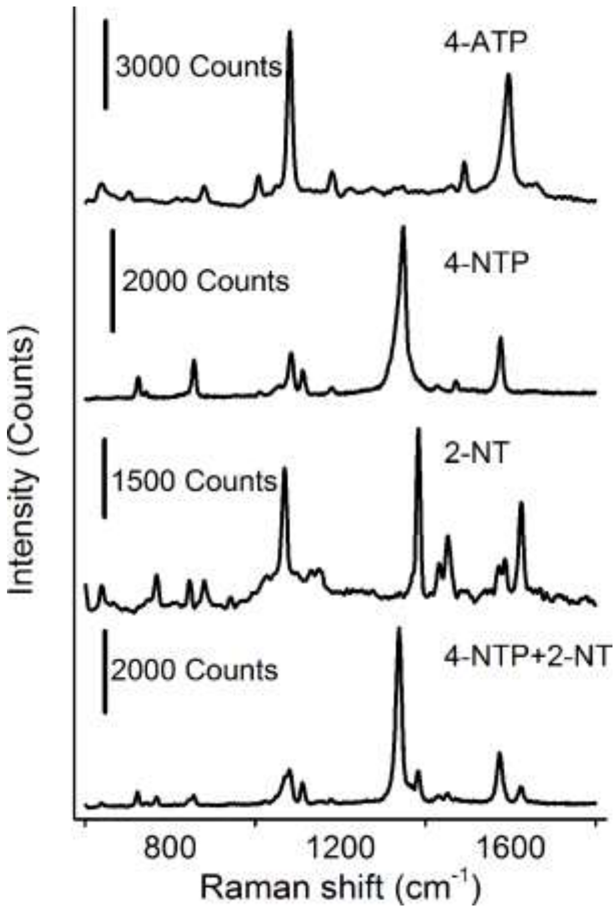

Figure S9. SERS spectra of 4-aminothiophenol (4-ATP), 4-nitrothiophenol (4-NTP), 2-naphthalenethiol (2-NT), and a mixture of 4-NTP and 2-NT on a GNP monolayer electrostatically adsorbed on the surface of an amine-functionalized Si wafer. The spectra were vertically offset for clarity. The signal integration time per spectrum was 10 s and the laser wavelength was 638 nm.

Table S2. SERS vibrational modes of 2-NT and their band assignments[6]

Raman shift (cm^{-1})	Band assignment
639	*δ*(CC)
767	*δ*(CC)
845	*δ*(CH)
940	*δ*(SH)
1067	*δ*(CH)
1378	*v*(CC)
1448	*v*(CC)
1619	*v*(CC)

v: stretching vibration, *δ*: deformation vibration

Table S3. SERS vibrational modes of 4-ATP and their band assignments[7]

Raman shift (cm^{-1})	Band assignment
635	*δ*(CCC)
1006	*δ*(CC) + *δ*(CCC)
1080	*v*(CS)
1178	*δ*(CH)
1590	*v*(CC)

v: stretching vibration, *δ*: deformation vibration

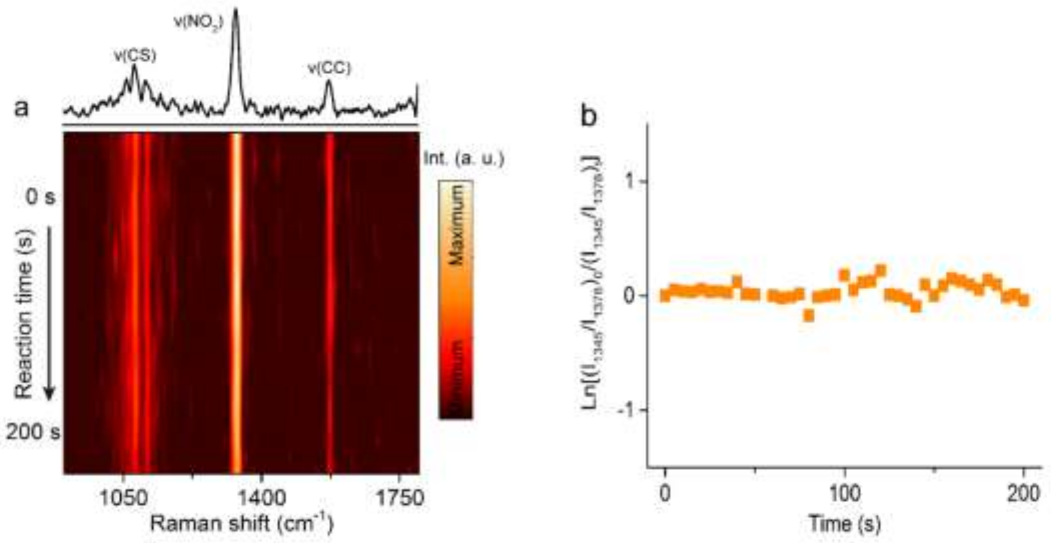

Figure S10. (a) Time-resolved SERS spectra of a mixture of 4-NTP and 2-NT immobilized on the S-GNP surface and enhanced by a single GNP dimer structure without borohydride addition. (b) Time-resolved SERS intensities of the 1345 cm^{-1} band (v(NO_2) of 4-NTP) relative to the 1378 cm^{-1} peak of 2-NT.

References:

[1] J. Turkevich, P. C. Stevenson and J. Hillier, *Discuss. Faraday Soc.*, 1951, **11**, 55-75.

[2] G. Frens, *Nature*, 1973, **241**, 20-22.

[3] H. Cha, J. H. Yoon and S. Yoon, *ACS Nano*, 2014, **8**, 8554-8563.

[4] A. Martín, A. Pescaglini, C. Schopf, V. Scardaci, R. Coull, L. Byrne and D. Iacopino, *J. Phys. Chem. C*, 2014, **118**, 13260-13267

[5] X. M. Yang, D. A. Tryk, K. Hasimoto, A. Fujishima1, *Appl. Phys. Lett*., 1996, **69**, 4020.

[6] A. N. Severyukhina, B. V. Parakhonskiy, E. S. Prikhozhdenko, D. A. Gorin, G. B. Sukhorukov, H. Möhwald and A. M. Yashchenok, *ACS Appl. Mater. Interfaces*, 2015, **7**, 15466-15473.

[7] K. Kim and J. K. Yoon, *J. Phys. Chem. B*, 2005, **109**, 20731-20736.
